# How are the domains of women's inclusion, justice, and security associated with maternal and infant mortality across countries? Insights from the Women, Peace, and Security Index

**DOI:** 10.1016/j.ssmph.2019.100486

**Published:** 2019-11-20

**Authors:** Jeni Klugman, Li Li, Kathryn M. Barker, Jennifer Parsons, Kelly Dale

**Affiliations:** a Institute for Women, Peace and Security, Georgetown University, 1412 36th Street, N.W., Washington, DC, 20057, United States; b Hutchinson Institute for Cancer Outcomes Research, Fred Hutchison Cancer Research Center, 1100 Fairview Ave. N., Seattle, WA, 98109, United States; c Center on Gender Equity and Health, Division of Infectious Diseases and Global Public Health, University of California San Diego, 9500 Gilman Drive, La Jolla, CA, 92093, United States

**Keywords:** Inclusion, Index, Justice, Infant mortality, Maternal mortality, Women's health

## Abstract

Women's autonomy and empowerment in their homes, communities, and societies at large have been shown, through many direct and indirect pathways, to be associated with maternal and infant health. A novel global measure—the Women, Peace, and Security (WPS) Index—that bridges insights from gender and development indices with those from peace and security has recently been developed to capture the constructs of women's inclusion, justice, and security, using indicators and targets in the Sustainable Development Goals. This paper adds to the growing literature about the importance of gender inequality to key mortality outcomes for women and children by investigating the associations between nations' WPS Index scores and maternal mortality ratios and infant mortality rates. We use a range of international databases to obtain country-level data from 144 nations on health, demographic, income, and gender equality indicators. The aim is to highlight the role of women's inclusion, justice, and security in explaining national rates of maternal and infant mortality. Fully adjusted Poisson regression models indicate that a one point (0.01) increase on the WPS Index score is associated with a 2.0% reduction in the number of maternal deaths and a 2.3% reduction in the number of infant deaths. For a country such as Sierra Leone, with a maternal mortality ratio of 1360 maternal deaths per 100,000 live births, a one point improvement in the WPS Index would correspond to a maternal mortality ratio of 1,332, or 28 fewer deaths per 100,000 births. These associations are ecological and apply to the average level of mortality at the country level rather than the likelihood or risk faced at the individual level. Although we cannot claim causality for the observed relations in the cross-country regressions, the findings and recurring patterns are both suggestive and encouraging about the potential contributions of women's inclusion, justice, and security to maternal and infant mortality.

## Highlights

• The Women, Peace, and Security (WPS) Index is a novel composite index, developed in 2019.
• The index measures women’s inclusion (economic, political, social); justice; and security (at home and in society).
• Using data from 144 nations, we find a significant relationship between the WPS Index and maternal and infant mortality.
• The inclusion and security aspects of the WPS Index are especially significant.

## 1 Introduction

Although global trends in key health outcomes over recent decades are encouraging, there is growing recognition of the deep-seated structural barriers to their improvement, including in gender inequality. The targets and indicators agreed upon by the 193 countries in the Sustainable Development Goals (SDGs) illustrate that health equity (SDG 3) is not a stand-alone goal and underlie the synergies between health outcomes and gender equality (SDG 5), economic opportunities (SDG 8), and ensuring peaceful societies (SDG 16), among others (WHO, 2017). The targets to reduce maternal and infant mortality and the goal to achieve gender equality and empower all women and girls provide further impetus to investigate the connections between these important agendas.

This paper focuses on two key health outcomes used to measure progress against SDG 3—maternal mortality ratio (MMR) and infant mortality rate (IMR). These health metrics are widely used as measures of country progress and are important indicators of population health and socioeconomic development generally (Gruber, Hendren, & Townsend, 2014, Reidpath & Allotey, 2003). The global maternal mortality ratio has declined by 37% since 2000, yet in 2015, 303,000 women around the world died because of complications during pregnancy or childbirth, largely from preventable causes, the vast majority in developing countries (Alkema et al., 2016). Infant mortality has also been declining, but still totaled 4.2 million infant deaths in 2016 (WHO, 2018a).

A number of studies have investigated the correlates of maternal and infant mortality outcomes. These correlates range from distal factors, such as the level of national income, to more proximate factors, such as the presence of skilled birth attendants during labor. Although a detailed review of these studies is beyond the scope of this article, a brief review is presented here.

Empirical studies suggest that per capita income is an important determinant of child and maternal mortalities globally (Grépin & Klugman, 2013) and regionally, in sub-Saharan Africa (Ashiabi, Nketiah-Amponsah, & Senadza, 2016; Buor & Bream, 2004) and the Eastern Mediterranean (Global Burden of Disease Eastern Mediterranean Region Maternal Mortality Collaborators, 2018). Higher incomes increase people's capacity to demand higher quality healthcare (Cutler, Deaton, & Lleras-Muney, 2006) and the capability of governments to finance access to quality services (Ashiabi et al., 2016). Poverty is a risk factor for maternal mortality (Ronsmans & Graham, 2006; Shiffman, 2000; Victora et al., 2010) and infant mortality (Filmer & Pritchett, 1997; Houweling, Kunst, Looman, & Mackenbach, 2005). Much of the effect appears to be due to poverty's role in limiting access to care (Girum & Wasie, 2017; Grépin & Klugman, 2013; Muldoon et al., 2011), which is compounded by living a longer distance to the nearest health clinic (WHO, 2016; Montgomery, Ram, Kumar, & Jha, 2014). Women who live in poverty may face higher mortality risks related to nutrition and to communicable and noncommunicable diseases directly related to lack of resources (United Nations, 2009). Studies have also examined how government health expenditures are associated with MMR, including the World Health Organization (WHO) systematic review of maternal mortality and morbidity (Bertrán et al., 2005), which found increased health expenditures per capita were a statistically significant factor in countries with lower maternal mortality ratios. In 44 sub-Saharan African countries, public and private healthcare spending was associated with lower infant mortality (Novignon, Olakojo & Nonvignon, 2012).

While money clearly matters, the evidence also points to a broader range of social factors driving maternal and infant health outcomes. For example, Schell, Reilly, Rosling, Peterson, and Mia Ekström (2007) found that in low-income countries, female literacy was more important than income per capita for infant mortality. A 2011 global study found that, compared with women with more than 12 years of education, women with between one and six years of education had twice the risk of maternal mortality, while those with no education had nearly triple the risk (Karlsen et al., 2011).

Adolescent fertility is positively correlated with higher maternal (Blanc, Winfrey, & Ross, 2013) and infant mortality rates (Chen et al., 2007; Hajizadeh, Nando, & Heymann, 2014), although estimates of the size of the increased risk vary greatly (Nove, Matthews, Neal, & Camacho, 2014). The WHO identified the presence of a skilled birth attendant as a significant factor in reducing maternal mortality (Betrán, Wojdyla, Posner, & Gülmezoglu, 2005). A 2018 study of 47 Muslim-majority countries found that skilled birth attendants had a significant positive impact on rates of infant mortality (Akseer et al., 2018).

Water and sanitation are also important correlates of MMR and IMR. In a study of 193 countries, Cheng, Schuster-Wallace, Watt, Newbold, and Mente (2012) show that access to water and improved sanitation are correlated with lower infant mortality. Improved handwashing and sanitation practices during childbirth have been shown to reduce the risk of infections, sepsis, and death for infants and mothers by up to 25% (Blencowe et al., 2011).

Recognizing the breadth and multiple levels of factors that influence maternal and infant mortality, a number of researchers have utilized composite measures to test the association between the multiple factors of influence and health outcomes across countries. These studies employ an ecological study design, with the country as the unit of analysis, to assess the association between female empowerment indices and a range of factors, such as income per capita and health service provision. One composite measure is the United Nations (UN) Development Programme Gender Inequality Index (GII), which aggregates gaps between men and women in labor force participation and empowerment (secondary education and parliamentary seats), as well as MMR and adolescent fertility. Using the 2010 GII, Brinda, Rajkumar, and Enemark (2015) found a positive association between gender inequality and infant mortality rates, in an analysis covering 135 countries. Lan and Tavrow (2017) used two other composite measures, the Gender Equity Index (GEI), which measures the gaps between women and men in education, the economy, and political representation, and the Social Institutions and Gender Index (SIGI), which measures discrimination against women in social institutions (formal and informal laws, social norms, and practices), and found that both were significantly correlated with maternal mortality in 44 low-income countries, although none of the indices were consistently significant.

The present study builds upon the existing empirical evidence by undertaking an ecological analysis using a novel global composite measure: the Women, Peace, and Security (WPS) Index. In addition to being the first gender index framed explicitly by the Sustainable Development Agenda, and selecting indicators agreed upon in the Sustainable Development Goals, the WPS Index is also the first to bring together women's inclusion, justice, and security into a single number and ranking (Klugman, 2019). The WPS Index aggregates measures of women's inclusion (economic, social, political), justice (formal laws and informal discrimination), and security (family, community, and societal levels). Existing gender indices—such as the World Economic Forum's Gender Gap Index—are typically limited to such aspects as whether women have completed secondary school or are engaged in paid work (Klugman, 2019). These aspects of inclusion are undoubtedly important but are incomplete in the absence of aspects of justice and security. The WPS Index responds to this gap by incorporating several indicators that have never been used in other prominent gender indices: women's perceptions of safety in the community and organized violence; whether women's paid work is deemed acceptable by men in the society; cell phone use; a bias for sons; and intimate partner violence (Klugman, 2019). The WPS Index provides a useful summary measure of aspects of both women's status (e.g., whether they are legally discriminated against in the society and whether they are in positions of political leadership), as well as aspects of their well-being (safety in their own homes and community, engagement in paid work, and their educational attainment). The WPS Index does not, however, directly capture gaps in women's achievements compared with men; it is not a measure of gender equality or inequality.

The WPS Index has several advantages for the present purposes,^1^ including that it is not endogenous (i.e., it does not include MMR and IMR). It is based on internationally comparable data from published sources rather than expert assessment of achievement, which is the case for elements of the SIGI and World Economic Forum measures, and the construct is easy to understand.^2^ Further attractions include its policy relevance, as the components are generally actionable (e.g., expanding access to education for girls and eliminating legal discrimination) and the fact that the index is recently available for a large number of countries (n=153) and that nations’ scores will be updated every two years (Georgetown Institute for Women, Peace and Security and Peace Research Institute Oslo, 2017, p. 10).

To advance our knowledge, we undertake ecological investigations using the WPS Index and average level of maternal and infant mortality at the country level rather than the “risk” faced at the individual level. Although we cannot claim causality for the observed relations in the cross-country regressions, the findings and recurring patterns are both suggestive and encouraging about the potential role of women's status.

## 2 Methods

### 2.1 Data

A range of data sources are used to obtain the country-level variables used in analysis. These data sources include national Demographic and Health Surveys (DHS), Gallup Polls, and surveys from the World Bank and UN Member Organizations and their partners (e.g., the International Labor Organization (ILO), the United Nations Educational, Scientific, and Cultural Organization (UNESCO), the United Nations Children's Fund (UNICEF), and the United Nations Entity for Gender Equality and the Empowerment of Women (UN Women). The list of variables, associated definitions, sources, and years are summarized in Table 1. As shown, the World Development Indicators dataset—compiled by the World Bank using the most currently accurate national, regional, and global estimates available (World Bank, 2018)—was used for the health-dependent variables (MMR and IMR) as well as national income and health expenditure per capita, improved sanitation and water source, rural population share, poverty headcount rate at $1.90 per day, adolescent fertility rate, and HIV prevalence. The World Governance Indicators dataset was used for political stability and government effectiveness variables, and WHO data was used for private health expenditure and physician data. Antenatal care (at least one visit) and skilled birth attendance are from Demographic and Health Surveys (DHS) Multiple Indicator Cluster Survey (MICS), and other nationally representative sources.

**Table 1:** Variables and sources of data.

Variable	Definition	Source	Year(s)
MMR	Maternal mortality ratio (modeled estimate, per 100,000 live births)	World Bank World Development Indicators	2015
IMR	Infant mortality rate (per 1000 live births)	World Bank World Development Indicators	2015
*Women, Peace, and Security (WPS) Index and Sub-indices*			
WPS Index	A composite index measuring women's achievements for the three dimensions of inclusion, justice, and security	WPS Index	2017
*WPS Inclusion Sub-index*			
Education	Average number of years of education completed by women aged ≥25 years	UNESCO Institute for Statistics	
Financial inclusion	The percentage of women aged ≥15 years who reported having an account alone or jointly at a bank or other type of financial institution or personally using a mobile money service	World Bank Global Findex Database	
Employment	The percentage of a country's female population aged ≥25 years that is employed.	ILOSTAT database	
Cell phone use	The percentage of women aged ≥15 years responding “Yes” to the Gallup World Poll question: “Do you have a mobile phone that you use to make and receive personal calls?”	Gallup World Poll 2016	
Parliamentary seats	The percentage of seats held by women in lower and upper houses of national parliaments.	Inter-Parliamentary Union	
*WPS Justice Sub-index*			
Legal discrimination	Aggregate score of laws and regulations that limit women's ability to participate in the society or economy or that differentiate between men and women, as measured by Women, Business, and the Law	World Bank, Women, Business, and the Law database	
Son bias	Sex ratio at birth (ratio of male births to female births). An excess number of births of boys over girls relative to demographic norms (ratio of 1.05 boys to 1.00 girls) reflects discrimination against girls and women	United Nations Department of Economic and Social Affairs, 2016. 2015 Revision of the World Population Prospects	
Discriminatory norms	Percentage of men aged ≥15 years who responded “No” to the Gallup World Poll question: “Is it perfectly acceptable for any woman in your family to have a paid job outside the home if she wants one?”	Gallup, Inc., and International Labour Organization 2017. Towards a Better Future for Women and Work: Voices of Women and Men.	
*WPS Security Sub-index*			
Lifetime intimate partner violence	The percentage of women who have experienced physical or sexual violence committed by their intimate partner	UN Women Global Database on Violence against Women; DHS (Demographic and Health Surveys) Program STATcompiler database 2016 and United Nations Population Fund (UNFPA) Asia-Pacific.	
Perception of community safety	Percentage of women aged ≥15 years who responded “Yes” to the Gallup World Poll question: “Do you feel safe walking alone at night in the city or area where you live?”	Gallup World Poll 2016	
Organized violence	Total number of battle deaths from state-based, non–state based, or one-sided conflicts per 100,000 people	UCDP (Uppsala Conflict Data Program). UCDP Georeferenced Event Dataset	
*National Income/Health Expenditure*			
Real gross domestic product (GDP) per capita	GDP per capita (constant, 2010 US$)	World Bank World Development Indicators	2010–2016
Real health expenditure per capita	Health expenditure per capita, PPP (constant 2011 international $)	World Bank World Development Indicators	2014
Out of pocket expenditure per capita	Out-of-pocket expenditure per capita in PPP international $	World Health Organization data repository	2011–2015
Health expenditure GDP share	Health expenditure, total (% of GDP)	World Bank World Development Indicators	2014
*Government*			
Political stability	Political stability and absence of violence/terrorism measures perceptions of the likelihood of political instability and/or politically motivated violence	Worldwide Governance Indicators	2013–2016
Government effectiveness	Perceptions of the quality of public services, quality of the civil service and the degree of its independence from political pressures, and quality of policy formulation and implementation and the credibility of government commitment to such policies	Worldwide Governance Indicators	2013–2016
*Water/Sanitation*			
Improved water source	Improved water source (% of population with access)	World Bank World Development Indicators	2011–2015
Improved sanitation facilities	Improved sanitation facilities (% of population with access)	World Bank World Development Indicators	2011–2015
*Access to Health Care*			
Physician density	Physicians density (per 1000 population)	World Health Organization data repository	1997–2016
Antenatal care (%), at least one visit	Percentage of women (aged 15–49 years) attended at least once during pregnancy by skilled health personnel (doctor, nurse, or midwife) and the percentage attended by any provider at least once	UNICEF State of the World's Children: DHS, MICS and other nationally representative sources.	2011–2016
Skilled birth attendant (%)	Percentage of births attended by skilled heath personnel (doctor, nurse, or midwife)	UNICEF State of the World's Children: Joint UNICEF/WHO SBA database, November 2017 update, based on DHS, MICS and other nationally representative sources.	2013–2016
*Population*/*Poverty Line*			
Rural Population Share	Rural population (% of total population)	World Bank World Development Indicators	2011–2016
Poverty head count ratio at $1.90 per day	Poverty headcount ratio at $1.90 per day (2011 PPP) (% of population)	World Bank World Development Indicators	2008–2015
*Family*			
Adolescent fertility rate	Adolescent fertility rate (births per 1000 women, aged 15–19 years)	World Bank World Development Indicators	2015
*Disease*			
HIV prevalence	Prevalence of HIV, total (% of population aged 15–49 years)	World Bank World Development Indicators	2015

We recognize at the outset that the data are not perfect. Although gender-disaggregated data are more available in the health sphere (relative to the environment sector, for example), gaps in availability and quality affect the indicators used for cross-country analysis. This study draws on the best available estimates from internationally comparable sources, bearing these caveats in mind.

### 2.2 Measures

Study outcome measures are MMR and IMR. MMR measures the number of women who die during pregnancy, childbirth, or the six weeks after delivery per 100,000 live births in a given region (WHO, 2018b). IMR measures the number of deaths per 1000 live births of children under one year of age (WHO, 2018b).

The primary exposure of interest is the WPS Index, with a theoretical range from 0 to 1, with a ranking of 1 indicating a perfect national score on inclusion, justice, and security. Formative research for the selection of final indicators included in the index was based on extensive review of the academic literature and reports by the United Nations. All indicators included in the index are explicit aspects of the SDGs. Development of the index and final indicator selection criteria has been described in-depth elsewhere (Georgetown Institute for Women, Peace and Security and Peace Research Institute Oslo, 2017; Klugman, 2019). The index and scores for 153 countries were published for the first time in October 2017 (Georgetown Institute for Women, Peace and Security and Peace Research Institute Oslo, 2017; Klugman, 2019). The 153 countries represent more than 98% of the world's population across all levels of income and development.^3^ Scores for the present analysis use the 2017 country rankings (provided in Appendix A). The index contains three sub-indices: *inclusion* (economic, social, and political spheres); *justice* (formal laws and informal discrimination); and *security* (at the individual, community, and societal levels). The WPS Index scores are generated using the variables listed below in Table 1. In addition to the WPS Index and sub-indices, a range of covariates shown in empirical literature to be correlated with both maternal and infant mortality are examined for inclusion in final analyses.

### 2.3 Analysis

The process for final model selection follows four steps. We begin by examining bivariate correlations between the variables that the literature suggests are significant determinants of infant and maternal mortality and our outcome measures (MMR and IMR), using the threshold of a correlation of ±0.60 (i.e., moderate to high correlation) to determine inclusion (Table 2) (Hinkle, Wiersma, & Jurs, 2009). For both MMR and IMR, the following variables emerged as having moderate to high correlation and are retained for subsequent analysis: real GDP per capita, real health expenditure per capita, out-of-pocket expenditure per capita, government effectiveness, improved water source, improved sanitation facilities, physician density, antenatal care, presence of a skilled birth attendant, rural population share, poverty headcount ratio, and adolescent fertility rate. HIV prevalence was included for MMR but not for IMR (Spearman's correlation of ρ=0.607 and 0.536, respectively).

**Table 2:** Bivariate Spearman correlations with MMR and IMR.

	(1)	(2)	(3)	(4)
	MMR	N	IMR	N
**National Income and Health Expenditure**				
Real GDP per capita	−0.857 (p < 0.001)	178	−0.870 (p < 0.001)	189
Real health expenditure per capita	−0.861 (p < 0.001)	179	−0.885 (p < 0.001)	190
Out-of-pocket expenditure per capita	−0.799 (p < 0.001)	179	−0.776 (p < 0.001)	190
Health expenditure share GDP	−0.343 (p < 0.001)	179	−0.361 (p < 0.001)	190
**Government**				
Political stability	−0.539 (p < 0.001)	181	−0.564 (p < 0.001)	191
Government effectiveness	−0.782 (p < 0.001)	181	−0.815 (p < 0.001)	191
**Water/Sanitation**				
Improved water source	−0.846 (p < 0.001)	178	−0.870 (p < 0.001)	187
Improved sanitation facilities	−0.895 (p < 0.001)	177	−0.872 (p < 0.001)	185
**Access to Health Care**				
Physician density, per 1000 population	−0.876 (p < 0.001)	174	−0.846 (p < 0.001)	185
Antenatal care (%), at least one visit	−0.646 (p < 0.001)	154	−0.677 (p < 0.001)	161
Skilled birth attendant (%)	−0.848 (p < 0.001)	156	−0.797 (p < 0.001)	164
**Population/Poverty Line**				
Rural population share	0.647 (p < 0.001)	181	0.636 (p < 0.001)	192
Poverty headcount ratio at $1.90/day	0.883 (p < 0.001)	143	0.857 (p < 0.001)	145
**Family**				
Adolescent fertility rate	0.827 (p < 0.001)	181	0.803 (p < 0.001)	183
**Disease**				
HIV prevalence	0.607 (p < 0.001)	132	0.536 (p < 0.001)	132
**WPS**				
WPS Index	−0.795 (p < 0.001)	153	−0.820 (p < 0.001)	153
WPS Inclusion Sub-index	−0.792 (p < 0.001)	153	−0.826 (p < 0.001)	153
WPS Justice Sub-index	−0.5171 (p < 0.001)	153	−0.597 (p < 0.001)	153
WPS Security Sub-index	−0.6374 (p < 0.001)	153	−0.592 (p < 0.001)	153

Abbreviations: GDP, gross domestic product; IMR, infant mortality rate; MMR, maternal mortality ratio; WPS, Women, Peace, and Security Index.

NB: Sample sizes reflect existing data for each variable.

We next examine the correlation between these remaining variables and the WPS Index (Table 3). Given our exposure of interest is the WPS Index, any covariates with high correlation (±0.80) with the WPS Index are removed. Only government effectiveness had a high correlation with the WPS Index and was subsequently removed.

**Table 3:** Bivariate Spearman correlations with WPS index.

	WPS Index	N
**National Income and Health Expenditure**		
Real GPD per capita	0.785 (p < 0.001)	150
Real health expenditure per capita	0.785 (p < 0.001)	152
Out-of-pocket expenditure per capita	0.702 (p < 0.001)	152
**Government**		
Government effectiveness	0.810 (p < 0.001)	153
**Sanitary/Environmental**		
Improved water source	0.734 (p < 0.001)	152
Improved sanitation facilities	0.720 (p < 0.001)	151
**Access to Health Care**		
Physician density, per 1000 population	0.732 (p < 0.001)	149
Antenatal care (%), at least one visit	0.617 (p < 0.001)	127
Skilled birth attendant (%)	0.703 (p < 0.001)	130
**Population/Poverty Line**		
Rural population share	−0.544 (p < 0.001)	153
Poverty headcount ratio at $1.90/day	−0.704 (p < 0.001)	131
**Family**		
Adolescent fertility rate	−0.689 (p < 0.001)	153
**Disease**		
HIV prevalence*	−0.266 (p < 0.001)	118

Abbreviations: GDP, gross domestic product; WPS, Women, Peace, and Security Index.

NB: Sample sizes reflect existing data for each variable.

*Reached correlation threshold only with MMR.

Stepwise Poisson regression is conducted with the remaining covariates. Given the high collinearity between variables, when included in models together, the direction of the relationships between some of the covariates and the outcome variables changes (real health expenditure per capita, out-of-pocket expenditure per capita, rural population, and antenatal care). In the third step, we look for high correlation (±0.80) between the remaining twelve covariates. Through this process, we find that the log of real health expenditure per capita and log of gross domestic product (GDP) per capita are highly correlated (*r*=0.96); poverty headcount and total sanitation are highly correlated (ρ=−0.82). Therefore, we exclude real health expenditure per capita and poverty head count from subsequent analyses.

In the final step, we conduct stepwise Poisson regression with MMR and IMR as outcome variables. Final covariates included in MMR models are GDP per capita, physician density, presence of a skilled birth attendant, adolescent fertility rate, and HIV prevalence. Final covariates in IMR models are GDP per capita, improved water source, access to improved sanitation facilities, physician density, and adolescent fertility rate.

The final number of country observations in the full models is 105 countries for maternal mortality and 144 countries for infant mortality. All analyses are conducted in Stata/SE 15.1.

## 3 Results

Table 4 shows the summary statistics of the two dependent variables and seven independent variables. There is large variation across countries. MMRs range from 3 (Finland, Greece, Iceland, and Poland) to 1360 (Sierra Leone) per 100,000 live births, while IMR ranges from 1.5 (Luxembourg) to 96 (Angola) per 1000 live births.

**Table 4:** Descriptive statistics for dependent and independent variables.

	N	Mean	SD	Min	Max
Maternal mortality ratio	144	168.76	236.06	3.00	1360.00
Infant mortality rate	144	23.67	22.69	1.50	96.00
Women, Peace, and Security Index	144	0.69	0.11	0.38	0.89
Real gross domestic product per capita	144	14351.80	20270.22	218.28	108600.90
Improved water source	144	88.90	14.29	49.00	100.00
Improved sanitation facilities	144	72.91	29.31	11.60	100.00
Physician density	144	1.74	1.49	0.02	6.26
Skilled birth attendant	122	84.35	20.10	20.20	100.00
Adolescent fertility rate	144	47.03	40.24	2.84	173.74
HIV prevalence	114	2.13	4.86	0.10	27.20

*Data availability is different for the skilled birth attendant and HIV prevalence variables.

Large differences in maternal and infant mortality are seen across regions (Table 5). In Europe and Central Asia, MMR (13.5) and IMR (7.3) are 36 times and 7 times, respectively, lower than in sub-Saharan Africa, where MMR is 481.1 per 100,000 live births and IMR is 52.7 per 1000 live births.

**Table 5:** Descriptive statistics for dependent and independent variables’ mean, by region and income group.

Region	N	MMR	IMR	WPS Index	Real GDP Per Capita	Improved Water Source	Improved Sanitation Facilities	Physician Density	Skilled Birth Attendant*	Adolescent Fertility Rate	HIV Prevalence*
East Asia and Pacific	13	69.9	14.8	0.7	15244.5	91.4	80.8	1.6	90.0	25.9	0.4
Europe and Central Asia	46	13.5	7.3	0.8	26766.1	97.8	95.4	3.3	99.3	15.7	0.3
Latin America and Caribbean	22	96.0	17.8	0.7	7155.2	91.9	79.7	1.4	91.9	62.9	0.7
Middle East and North Africa	15	61.9	13.8	0.6	18560.9	93.3	90.9	1.9	89.6	25.2	0.1
North America	2	10.5	5.0	0.8	51313.2	99.5	99.9	2.6	99.2	15.3	0.5
South Asia	8	178.5	34.1	0.6	2552.9	89.2	60.6	1.0	70.9	40.9	0.2
Sub-Saharan Africa	38	481.1	52.7	0.6	2062.2	73.2	33.1	0.2	67.3	94.6	5.8

Abbreviations: GDP, gross domestic product; IMR, infant mortality rate; MMR, maternal mortality ratio; WPS, Women, Peace, and Security Index.

* Data availability is slightly different for the skilled birth attendant and HIV prevalence variables.

There is a similarly wide range in national achievements by region and by GDP per capita, access to improved water and sanitation facilities, physician density, skilled birth attendant, adolescent fertility rate, and HIV prevalence (Table 5). Europe, Central Asia, and North America perform the best across these indicators, while sub-Saharan Africa performs the worst.

### 3.1 Multivariate analysis

We proceed to investigate the statistical relationships between the WPS Index and MMR and IMR using Poisson regression. The first set of models (Table 6) examines null models with the WPS Index and each of the sub-indices. These models indicate that the WPS Index and each sub-index (inclusion, justice, and security) are significantly and negatively associated with mortality rates. For the overall index, the associated incidence rate ratio (IRR) for MMR is 0.9306, indicating that a one point (0.01) increase in a nation's WPS Index score is associated with a 7.0% reduction in the number of maternal deaths. The associated IRR for IMR for the WPS Index is 0.9433, indicating that a one point increase in a nation's WPS Index score is associated with a 5.7% reduction in the number of infant deaths. Among the three sub-indices, the inclusion sub-index has the greatest magnitude for both MMR and IMR.

**Table 6:** Women, peace, and security (WPS) Index and sub-indices on maternal mortality ratio and infant mortality rate.

Variables	(1)	(2)	(3)	(4)	(5)	(6)	(7)	(8)
	Maternal Mortality Ratio				Infant Mortality Rate			
	IRR (Coefficient) [SE]	IRR (Coefficient) [SE]	IRR (Coefficient) [SE]	IRR (Coefficient) [SE]	IRR (Coefficient) [SE]	IRR (Coefficient) [SE]	IRR (Coefficient) [SE]	IRR (Coefficient) [SE]
WPS Index	0.931 (−7.192***)				0.943 (−5.838***)			
	[0.0524]				[0.141]			
WPS Inclusion Sub-index		0.938 (−6.362***)				0.952 (−4.872***)		
		[0.0466]				[0.118]		
WPS Justice Sub-index			0.958 (−4.259***)				0.959 (−4.158***)	
			[0.0623]				[0.166]	
WPS Security Sub-index				0.958 (−4.292***)				0.967 (−3.358***)
				[0.0414]				[0.115]
Constant	1.102 (9.742***)	1.086 (8.236***)	1.090 (8.575***)	1.084 (8.058***)	1.072 (6.974***)	1.058 (5.632***)	1.068 (6.543***)	1.057 (5.498***)
	[0.0317]	[0.0207]	[0.0495]	[0.0272]	[0.0877]	[0.0558]	[0.132]	[0.0779]

Observations	153	153	153	153	153	153	153	153
Pseudo R2	0.430	0.504	0.106	0.223	0.429	0.481	0.156	0.197

Standard errors in brackets.

***p < 0.01, **p < 0.05, *p < 0.1.

The next set of models (Table 7) examine the relationship between GDP per capita, the WPS Index, and mortality rates. Increases in GDP per capita are often used to explain reductions in maternal and infant mortality. We find that when included in models separately, the IRRs for the WPS Index exceed those for log GDP per capita. When both variables are modeled together, both remain significant and negatively associated with infant and maternal mortality ratios, indicating that as WPS Index scores and GDP increase, mortality decreases. The coefficient sizes of GDP per capita decline once we include the WPS Index (Table 7, columns 2 to 3 and 5 to 6), suggesting that it is important to account for women's inclusion, justice, and security in understanding determinants of mortality.

**Table 7:** Results for multivariate regression model: GDP per capita and WPS index, maternal mortality ratio and infant mortality rate.

Variables	(1)	(2)	(3)	(4)	(5)	(6)
	Maternal Mortality Ratio			Infant Mortality Rate		
	IRR (Coefficient) [SE]	IRR (Coefficient) [SE]	IRR (Coefficient) [SE]	IRR (Coefficient) [SE]	IRR (Coefficient) [SE]	IRR (Coefficient) [SE]
WPS Index	0.925 (−7.830***)		0.972 (−2.874***)	0.939 (−6.332***)		0.971 (−2.979***)
	[0.0561]		[0.0805]	[0.150]		[0.218]
Log GDP per capita		0.992 (−0.812***)	0.993 (−0.676***)		0.995 (−0.548***)	0.996 (−0.399***)
		[0.00560]	(0.00689)		[0.0129]	[0.0172]
Constant	1.107 (10.14***)	1.121 (11.42***)	1.130 (12.19***)	1.076 (7.295***)	1.078 (7.552***)	1.086 (8.292***)
	[0.0340]	[0.0402]	[0.0456]	[0.0937]	[0.0980]	[0.111]

Observations	150	150	150	150	150	150
Pseudo R2	0.465	0.690	0.720	0.467	0.567	0.615

Abbreviations: GDP, gross domestic product; WPS, Women, Peace, and Security Index.

Standard errors in parentheses.

***p < 0.01, **p < 0.05, *p < 0.1.

In the final set of models, we include the remaining covariates. In the model examining maternal mortality (Table 8), we account for GDP per capita, improved sanitation, physician density, presence of a skilled birth attendant, adolescent fertility rate, and HIV prevalence (covariate selection process detailed above in the methods section). For infant mortality (Table 9), we account for GDP per capita, improved water and sanitation facilities, physician density, and adolescent fertility rate.

**Table 8:** Results for multivariate regression model: Maternal mortality ratio and WPS index—fully adjusted model.

Variables	Coefficient (SE)	IRR
WPS Index	−2.061***	0.980
	(0.0985)	
Log GDP per capita	−0.154***	0.998
	(0.0105)	
Improve sanitation facilities	−0.0107***	1.000
	(0.000508)	
Physician density	−0.480***	0.995
	(0.0189)	
Skilled birth attendant	−0.00134***	1.000
	(0.000417)	
Adolescent fertility rate	0.00395***	1.000
	(0.000228)	
HIV prevalence	0.0124***	1.000
	(0.00125)	
Constant	8.438***	1.088
	(0.0761)	
Observations	105	
Pseudo R2	0.855	

Abbreviations: GDP, gross domestic product; WPS, Women, Peace, and Security Index.

Standard errors in parentheses.

***p < 0.01, **p < 0.05, *p < 0.1.

**Table 9:** Results for multivariate regression model: Infant mortality rate and WPS index—fully adjusted model.

Variables	Coefficient	IRR
	(SE)	
WPS Index	−2.273***	0.978
	(0.248)	
Log GDP per capita	−0.136***	0.999
	(0.0251)	
Improved water source	−0.00335**	1.000
	(0.00161)	
Improve sanitation facilities	−0.00413***	1.000
	(0.00125)	
Physician density	−0.203***	0.998
	(0.0283)	
Adolescent fertility rate	0.00345***	1.000
	(0.000607)	
Constant	6.275***	1.065
	(0.184)	
		
Observations	144	
Pseudo R2	0.708	

Abbreviations: GDP, gross domestic product; WPS, Women, Peace, and Security Index.

Standard errors in parentheses.

***p < 0.01, **p < 0.05, *p < 0.1.

The inclusion of the known covariates attenuates the magnitude of the coefficients for the WPS Index, but the index remains statistically significant and negatively associated with both infant and maternal mortality. With respect to the covariates included in models, all associated coefficients are statistically significant, but the IRRs are very small. The largest IRR for the covariates is for physician density (MMR IRR = 0.9952; IMR IRR = 0.9980).

Fully adjusted Poisson regression models indicate that a one point (0.01) increase on the WPS Index score is associated with a 2.0% reduction in the number of maternal deaths (IRR = 0.9796), and a 2.3% reduction in the number of infant deaths (IRR = 0.9775). For a country such as Sierra Leone with a MMR of 1360 maternal deaths per 100,000 live births, a one point improvement in the WPS Index would correspond to an MMR of 1,332, or 28 fewer deaths per 100,000 births. For a country such as Angola with an IMR of 96 infant deaths per 1000 live births, a one point improvement in the WPS Index would correspond to an IMR of 93.8 infant deaths per 1000 live births, or 2.2 fewer infant deaths per 1000 live births.

## 4 Discussion

Our analysis takes advantage of recent improvements in data and innovations in measurement that generated the WPS Index. The WPS Index echoes the widely cited Human Development Index, with a focus on women, justice, and security. Compared with other composite gender indices, the WPS Index has the specific attraction—in the context of understanding drivers of health—of not being endogenous. For example, unlike the Gender Inequality Index, the WPS Index does not include commonly used health outcome indicators: MMR and IMR. The WPS Index also includes actionable components (for example, repealing legal discrimination against women) and is available for a large number of countries.

Our findings confirm significant positive associations between women's inclusion, justice, and security and maternal and infant mortality rates, after adjusting for the effects of major economic and health service variables. We find that a one point (0.1) increase on the WPS Index score is associated with a 2.0% reduction in the number of maternal deaths and a 2.3% reduction in the number of infant deaths. The WPS Index helps to explain more variation in MMR and IMR than GDP alone. This underscores that addressing exclusion, injustice, and a lack of security at home, in the community, and in society at large is not only important in itself, as reflected in the Sustainable Development Goals, but carries additional instrumental value to improving the health of mothers and infants.

Exploring the relationships between maternal and infant mortality and the WPS Index, we found that the inclusion sub-index had the largest magnitude of effect. This is perhaps unsurprising, as this dimension includes women's years of schooling, which has been shown in earlier studies to be influential (Gakidou, Cowling, Lozano, & Murray, 2010), as well as women's access to finance (savings and credit) and mobile phones. Although these variables may be important proxies for women's empowerment, it is also possible that access to finance can help women to cope with unexpected or catastrophic health events and thereby avert death. If a woman has her own financial account (as measured in the WPS Index), she may have more possibilities to access the health care needed for herself and her children. It is also possible that access to mobile technology—captured by the cell phone indicators in the inclusion dimension of the WPS Index—enables greater connectedness to information and services that can enable better health outcomes. A number of initiatives have sought to build on mobile subscriptions to pave the way for implementation of mobile health (mHealth) initiatives, especially among remote populations and in areas where women's mobility is limited, such as in Afghanistan (Yamin, Kaewkungwal, Singhasivanon, & Lawpoolsri, 2018). Further analysis of these potential mechanisms, beyond the use of mHealth messaging, would cast further light.

### 4.1 Limitations

First, this study presents cross-sectional analysis at the country level, and we cannot draw causal inferences from the results. Second, we need to avoid ecological fallacy, in which associations observed between variables on the aggregate level do not represent associations at the individual level. Third, the WPS Index is a new index and is currently only available for one point in time and is thus not able to be used to analyze and explain trends. An update is expected every two years, beginning at the end of 2019, which will facilitate further analysis. Fourth, although the WPS Index captures important domains indicative of women's status and achievements, further investigation of specific drivers is needed. Finally, our selection of indicators and measures is constrained by data availability. Although we used the most reliable and comparable data available, it is possible that some countries underreport mortality rates.

We undertake analysis that generated ecological associations and apply it to the average level of mortality in a population rather than the “likelihood” or “risk” faced at the individual level. Although we cannot claim causality for the observed relations in the cross-country regressions, and there are inevitable limitations in the quality of the data, the findings and recurring patterns are suggestive of and encouraging about the potential role of women's inclusion, justice, and security in advancing progress in maternal and infant mortality.

### 4.2 Conclusions

As underscored in the Sustainable Development Agenda, there are major synergies between health outcomes and gender equality. Our headline results underscore these synergies and the centrality of addressing gender inequality and women's empowerment as part of the Sustainable Development Agenda. Countries that performed the worst in terms of women's inclusion, justice, and security often have among the worst records in maternal and infant mortality. Our results point to the breadth of the Agenda and highlight specific levers that could be expected to accelerate future progress. Our results provide insights into the connections between the important international agendas called for in the SDGs, and maternal and infant mortality outcomes at a national level.

The SDGs include ambitious goals to reduce global maternal mortality and to end preventable deaths of newborns, alongside achieving gender equality and empowering all women and girls. To achieve these goals, it is imperative that the cross-cutting importance of women's status in society to achievements in health and economic development be addressed. As stated by Dr. Tedros Adhanom Ghebreyesus, Director General of the WHO, “…when women and girls are socially, economically and politically empowered, they are … more likely to control their sexuality and fertility, and more likely to be healthy. When women and girls have access to quality and comprehensive health care, information about their health and bodies, and the financial protection to be able to access services, it contributes to gender equality (Women Deliver, 2018).” The Goals have accelerated momentum to address the structural inequalities that impede the expansion of peace and prosperity. This paper buttresses that momentum by using a novel and robust measure showing the importance of women's wellbeing for the key health outcomes of maternal and infant mortality.

The breadth of the WPS Index and its significance to health outcomes illustrates the importance of a broad multipronged approach of policy and programmatic levers to advance women's well-being and to reduce maternal and infant mortality. By investigating the country-level data included in the WPS Index, policy makers can consider the program and policy reforms needed to enhance women's inclusion and security, and to address maternal and infant mortality.

## Ethical statement

Institutional Review Board deemed the nature of the research exempt from review, because of its sole use of publicly available secondary data sources.

## Figures and Tables

**Table:** 

Country	2017 WPS Index Value	Country	2017 WPS Index Value
Afghanistan	0.385	Lesotho	0.623
Albania	0.714	Liberia	0.588
Algeria	0.595	Lithuania	0.790
Angola	0.575	Luxembourg	0.841
Argentina	0.715	Madagascar	0.576
Armenia	0.654	Malawi	0.591
Australia	0.827	Malaysia	0.665
Austria	0.841	Maldives	0.605
Azerbaijan	0.623	Mali	0.505
Bahrain	0.709	Malta	0.795
Bangladesh	0.585	Mauritania	0.566
Belarus	0.767	Mauritius	0.705
Belgium	0.846	Mexico	0.686
Belize	0.682	Mongolia	0.761
Benin	0.582	Montenegro	0.770
Bhutan	0.628	Morocco	0.623
Bolivia (Plurinational State of)	0.707	Mozambique	0.628
Bosnia and Herzegovina	0.734	Myanmar	0.606
Botswana	0.656	Namibia	0.735
Brazil	0.677	Nepal	0.672
Bulgaria	0.735	Netherlands	0.854
Burkina Faso	0.609	New Zealand	0.826
Burundi	0.603	Nicaragua	0.717
Cambodia	0.660	Niger	0.538
Cameroon	0.548	Nigeria	0.583
Canada	0.854	North Macedonia	0.766
Central African Republic	0.474	Norway	0.879
Chad	0.551	Pakistan	0.441
Chile	0.713	Panama	0.694
China	0.671	Paraguay	0.696
Colombia	0.659	Peru	0.693
Comoros	0.583	Philippines	0.702
Congo	0.559	Poland	0.799
Costa Rica	0.730	Portugal	0.822
Côte d'Ivoire	0.604	Qatar	0.707
Croatia	0.804	Republic of Korea	0.800
Cyprus	0.802	Republic of Moldova	0.671
Czech Republic	0.797	Romania	0.739
Democratic Republic of the Congo	0.486	Russian Federation	0.721
Denmark	0.845	Rwanda	0.662
Dominican Republic	0.707	Saudi Arabia	0.655
Ecuador	0.746	Senegal	0.616
Egypt	0.559	Serbia	0.804
El Salvador	0.685	Sierra Leone	0.563
Estonia	0.809	Singapore	0.846
Eswatini	0.575	Slovakia	0.776
Ethiopia	0.633	Slovenia	0.861
Finland	0.855	Somalia	0.555
France	0.817	South Africa	0.732
Gabon	0.592	Spain	0.860
Georgia	0.727	Sri Lanka	0.656
Germany	0.845	Sudan	0.521
Ghana	0.701	Suriname	0.718
Greece	0.760	Sweden	0.854
Guatemala	0.650	Switzerland	0.871
Guinea	0.573	Syrian Arab Republic	0.385
Haiti	0.625	Tajikistan	0.687
Honduras	0.675	Thailand	0.670
Hungary	0.739	Togo	0.640
Iceland	0.886	Trinidad and Tobago	0.743
India	0.580	Tunisia	0.663
Indonesia	0.669	Turkey	0.634
Iran (Islamic Republic of)	0.619	Turkmenistan	0.679
Iraq	0.500	Uganda	0.654
Ireland	0.823	Ukraine	0.646
Israel	0.679	United Arab Emirates	0.746
Italy	0.790	United Kingdom	0.845
Jamaica	0.755	United Republic of Tanzania	0.672
Japan	0.798	Uruguay	0.714
Jordan	0.627	USA	0.810
Kazakhstan	0.741	Uzbekistan	0.720
Kenya	0.631	Venezuela (Bolivarian Republic of)	0.684
Kuwait	0.675	Viet Nam	0.665
Kyrgyzstan	0.690	Yemen	0.407
Lao People's Democratic Republic	0.723	Zambia	0.625
Latvia	0.787	Zimbabwe	0.697
Lebanon	0.547		

## Footnotes

1 We are grateful to Gary Darmstadt, Stanford University, for highlighting these benefits.

2 The WPS Index has the most in common with the SIGI Index and the Economist Intelligence Unit's Women's Economic Opportunity Index. However, those two indices rely extensively on expert judgment to measure various concepts or to address missing data and have many more indicators than the WPS Index. And the SIGI Index does not include economic dimensions, such as employment and cellphone use, or indicators of organized violence. The number of indicators in other gender indices currently available ranges from five (the GII, which includes maternal mortality) to 33 (SIGI Index) and averages around 16 indicators.

3 To be included in the WPS Index, a country must have data available for at least 8 of the 11 indicators. Of the 153 countries included in the WPS Index, 15 lacked data for 1 indicator, 8 lacked data for 2 indicators, and 8s lacked data for 3 indicators. Missing data were generally addressed by imputing the regional average for that score. In a few cases, the estimate for the country's nearest neighbor that shared common characteristics, such as level of development, was imputed. All these cases are footnoted in Statistical Table 1: https://giwps.georgetown.edu/wp-content/uploads/2017/10/WPS-Index-Report-2017-18.pdf.
